# Tuberculosis arthritis of ankle: A case report

**DOI:** 10.1002/ccr3.6112

**Published:** 2022-07-25

**Authors:** Babak Sayad, Arefeh Babazadeh, Sakineh Shabani, Rezvan Hosseinzadeh, Mohammad Barary, Soheil Ebrahimpour, Zeinab Mohseni Afshar

**Affiliations:** ^1^ Clinical Research Development Center Imam Reza Hospital, Kermanshah University of Medical Sciences Kermanshah Iran; ^2^ Infectious Diseases and Tropical Medicine Research Center Health Research Institute, Babol University of Medical Sciences Babol Iran; ^3^ Department of Radiology, School of Medicine Babol University of Medical Science Babol Iran; ^4^ Student Research Committee Babol University of Medical Sciences Babol Iran; ^5^ Student Research Committee, Virtual School of Medical Education and Management Shahid Beheshti University of Medical Sciences Tehran Iran; ^6^ Students' Scientific Research Center (SSRC) Tehran University of Medical Sciences Tehran Iran

**Keywords:** arthritis, infection, tuberculosis

## Abstract

Tuberculosis (TB) primarily involves the respiratory tract, but any organ in the body can be affected. In recent years, extrapulmonary TB cases have significantly increased due to the prevalence of immunocompromised patients. Here, we report a case of unilateral ankle arthritis due to *Mycobacterium tuberculosis* infection.

## Abstract 2

Although TB is rarely seen in developed countries, it is still a significant public health issue in developing countries. This infection primarily manifests with pulmonary involvements, but extrapulmonary TB signs and symptoms are also widely reported.

## 1 INTRODUCTION

Tuberculosis (TB) primarily involves the respiratory tract, but any organ in the body can be affected.^1^ In recent years, due to the prevalence of human immunodeficiency virus (HIV) infection and the widespread use of immunosuppressants in various settings, the prevalence of extrapulmonary TB manifestations has significantly increased,^2^ which may or may not be accompanied by active pulmonary involvement.^3^ Bone and joint involvement comprise up to 10% of extrapulmonary TB, approximately half of which are accompanied by pulmonary involvement.^4^, ^5^ In addition, bone and joint TB is divided into spinal and arthritic (synovial) diseases. Tuberculosis spondylitis has a significantly high prevalence, particularly in endemic areas. However, peripheral arthritic involvement has been rarely reported.^6^ Here, we report a case of unilateral ankle arthritis due to *Mycobacterium tuberculosis* infection.

## 2 CASE REPORT

**FIGURE 1:**
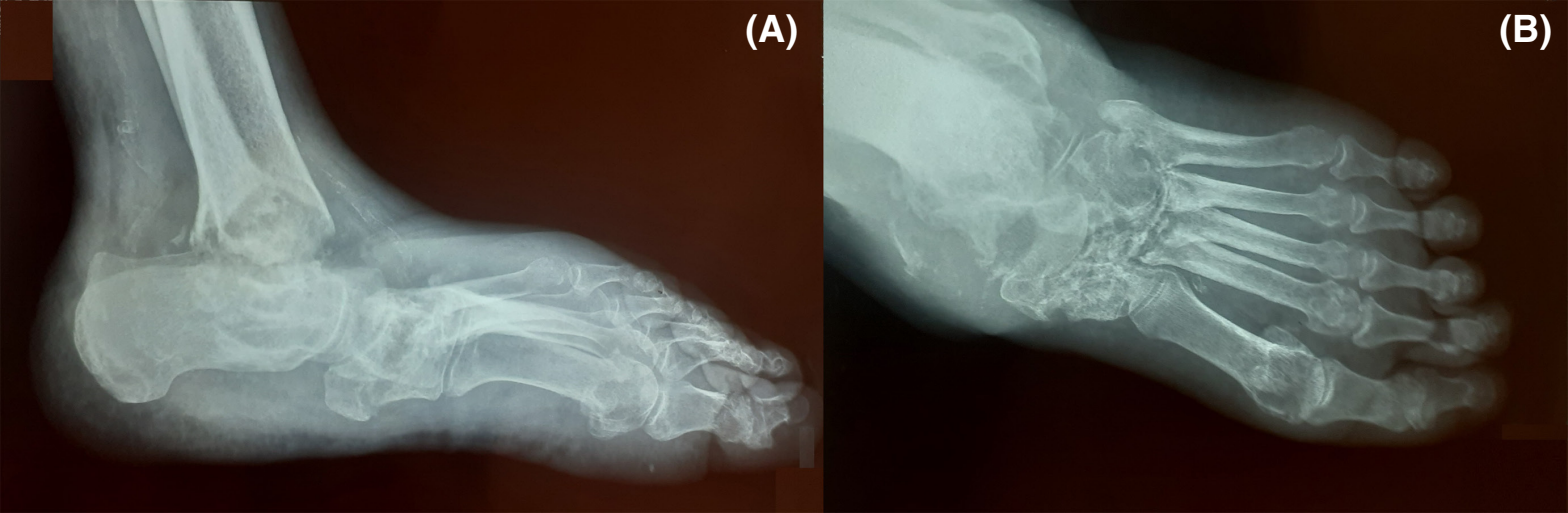
(A) Anteroposterior and (B) lateral X‐ray view of ankle and foot of the patient. Severe subchondral erosions and extensive destructive lesions in the ankle, intertarsal, and tarsometatarsal joints, along with periarticular osteoporosis and soft tissue swelling, are evident

A 90‐year‐old man presented to the infectious disease clinic with a draining ulcer on his left ankle. He has complained about this problem for the past 2 years, causing him to undergo various courses of antimicrobial treatment with no complete resolution. His past medical history was not significant, except for hypertension. On physical examination, tenderness, and induration, a fistulized ulcer with the discharge was detected on the affected ankle. Moreover, an obvious limitation of motion was evident in the joint. Abnormalities in his laboratory tests included an elevated erythrocyte sedimentation rate (ESR) (103 mm/h, reference value: < 30 mm/h) and C‐reactive protein (CRP) (28 mg/L, reference value: < 10 mg/L) levels, moderate anemia (hemoglobin = 10 g/dL, reference value: 13–17 g/dL), mild azotemia (creatinine = 1.5 mg/dL, reference value: < 1.2 mg/dL), and an active urine analysis (U/A) (WBC = 15–20, bacteria: many, nitrite: positive). The serologic evaluation was negative for brucellosis and viral markers, including HIV and hepatitis B (HBV) and C (HCV) viruses. The plain radiography (Figure 1) showed severe subchondral erosions and extensive destructive lesions in the left ankle, intertarsal, and tarsometatarsal joints and diffuse osteoporosis and periarticular soft tissue swelling.

**FIGURE 2:**
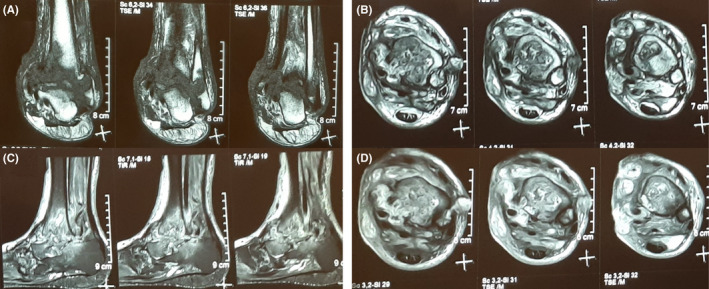
(A) coronal T1‐weighted, (B) axial T2‐weighted, (C) sagittal Short Tau inversion recovery (STIR), and (D) diffusion‐weighted (DW) magnetic resonance images (MRI) of ankle and foot of the patient. Severe cartilage loss and bone destruction in the ankle joint are evident. Also, bone marrow edema and intermediate to low‐signal lesions in the tibia and fibula with loculation in subcutaneous and extension to the skin can be observed. Moreover, the lesions are high signal in DW images

After an orthopedic surgery consultation, magnetic resonance imaging (MRI) of the affected foot and articular biopsy under an ultrasound guide were recommended (Figure 2). Although MRI was not performed as the patient did not consent, he gave his consent to undergo tissue sampling. After taking the biopsy, a chest high‐resolution computed tomography (HRCT) scan was performed, demonstrating hyperdense mediastinal lymph nodes, diffuse nodules, with a tree‐in‐bud pattern, almost in the right upper lobe, along with cicatricial atelectasis and fissure thickening in the right lung. All the mentioned findings were compatible with pulmonary tuberculosis (TB). Therefore, his sputum sample was drawn and sent for acid‐fast staining, culture, and Xpert MTB/RIF assay. Furthermore, the patient was started on quadruple antituberculosis treatment (isoniazid 300 mg daily, rifampin 600 mg daily, ethambutol 15 mg daily, and pyrazinamide 20 mg daily) with dose adjustment due to his mildly increased creatinine. The histopathology was indicative of granuloma formation compatible with tuberculosis. His polymerase chain reaction (PCR) test indicated rifampin‐sensitive *Mycobacterium tuberculosis*. Therefore, we continued anti‐TB treatment. His pain and swelling had improved significantly at one‐month follow‐up, and the discharge had stopped.

## 3 DISCUSSION

Tuberculosis arthritis occurs either as an infection spread from the adjacent bone or due to a hematogenous spread from a distant organ, such as the lungs.^7^ In non‐endemic areas, extrapulmonary TB usually occurs in an immunosuppression state, such as HIV, chronic diseases such as diabetes mellitus, alcoholism, and cancer, or being treated by corticosteroids or immunomodulators. Moreover, local injury, such as trauma, surgery, or intravenous drug use, can precipitate TB reactivation in the adjacent joints. However, none of the mentioned conditions might be present in endemic areas,^8^, ^9^, ^10^ and our patient did not have any of those risk factors.

The process of arthritic involvement by *Mycobacterium tuberculosis* is indolent and insidious that initially begins with simple synovitis, depicted by increased joint space in the imaging modalities. Then, granulation tissue formation, effusion, pannus formation, and cartilage destruction ensue. In the next stage, the underlying bone might be affected, or para‐articular cold abscesses form, which result in fistulae formation and draining sinus tract.^11^ Tuberculosis arthritis usually presents with a monoarticular pattern. Large and medium weight‐bearing joints such as the hip, and the knee, are the most common sites of involvement in peripheral TB arthritis.^12^ Nonetheless, a proportion present with foot or ankle joint involvements.^13^ In any subacute to chronic arthritis, we should consider tuberculosis as a potential differential diagnosis. This is especially true for those cases of arthritic involvement with a draining sinus tract to the overlying skin.^14^, ^15^

As happened to our patient, the diagnosis of TB arthritis is often delayed as it is repeatedly misdiagnosed as septic or reactive arthritis and treated accordingly.^15^ Therefore, a high index of suspicion is needed for timely detection. In order to confirm the diagnosis, the synovial fluid should be stained for acid‐fast bacteria (AFB), and a synovial biopsy should be drawn.^16^ Demonstration of granulomatous synovitis can indicate TB,^16^ as happened in our patient. However, as the diagnosis of TB arthritis is mainly based on clinical suspicion, and our patient had concomitant pulmonary findings indicative of TB, he was immediately started on antituberculosis treatment rather than waiting for the synovial biopsy results. Hence, detecting a simultaneous pulmonary TB can be a clue for tuberculosis as the cause of extrapulmonary involvement.^17^ The concomitant pulmonary involvement can be easily identified with imaging modalities such as chest X‐ray (CXR) or lung CT scan. However, sending sputum culture for PCR is mandated as some cases of pulmonary TB have normal pulmonary patterns on the imaging modalities.^18^ Moreover, culture and Xpert MTB/RIF assay are specific tools for identifying the isolate's antibiotic susceptibility.^19^ Fortunately, our patient had imaging abnormalities indicative of TB and positive sputum Xpert MTB/RIF assay results, indicating a rifampin‐sensitive isolate. Moreover, the histopathologic findings of his joint further confirmed our diagnosis.

Imaging modalities can also be beneficial in confirming TB arthritis suspicion. Plain radiography cannot aid the clinician in detecting the articular involvement early as the so‐called Phemister triad, including juxta‐articular osteoporosis, peripheral osseous erosions, and gradual joint space narrowing, is evident in later stages of TB arthritis.^20^ Nonetheless, our patient did have these findings on his plain radiography due to the delayed diagnosis. MRI is better for showing the associated abnormalities, such as joint effusion, loose bodies, and calcifications, but unfortunately, our patient did not consent to it.^12^

Finally, the treatment strategy for TB arthritis includes pharmacological therapy and surgical options in certain conditions. Medical therapy consists of the conventional four‐drug regimen for at least 9 months.^21^ However, a previous study concluded that concomitant use of antituberculosis drugs with bone debridement could significantly improve the patient’s outcome.^22^

## 4 CONCLUSION

Although TB is rarely seen in developed countries, it is still a significant public health issue in developing countries. This infection primarily manifests with pulmonary involvements, but extrapulmonary TB signs and symptoms are also widely reported, such as TB arthritis. When suspected, different imaging modalities (e.g., CXR) could help diagnose this condition, but further confirmation with molecular methods, such as PCR, is mandated. Moreover, culture and Xpert MTB/RIF assay could be beneficial in identifying the isolate's susceptibility to antibiotics. Then, a conventional four‐drug regimen for at least 9 months should be initiated for the patients, with further pharmacological and surgical options if indicated.

## AUTHOR CONTRIBUTIONS

**BS:** Data collection and writing the manuscript. **AB:** Data collection and helped with manuscript writing**. SS:** Data collection and writing the manuscript. **RH:** Visualization, helped with manuscript writing, and contributed substantial revisions to the manuscript's content. **MB:** Data collection, helped with manuscript writing, and contributed substantial revisions to the manuscript's content. **SE:** Data collection and writing the manuscript. **ZMA:** Design of the research study and supervision.

## CONFLICT OF INTEREST

All authors have no relevant financial interests to be declared.

## CONSENT

Written informed consent was obtained from the patient to publish the current case report.

## Data Availability

The data that support the findings of this study are available from the corresponding author upon reasonable request.
